# Anomalies in the Eruption of the Teeth of Man

**Published:** 1883-12

**Authors:** 


ARTICLE VIII.

### ABSTRACT OF A CLINICAL LECTURE BY DR. MAGITOT ON THE ANOMALIES IN THE ERUPTION OF THE TEETH OF MAN.

The anomalies in the eruption of the teeth are, as is well known, usually met with under one of the following terms :— 1. Congenital absence. 2. Early eruption of the milk teeth. 3. Late eruption of the permanent teeth.

The first form, *congenitnl absence*, may be divided into two varieties, the partial or total absence. The latter form has as yet been met with, except as a sequel to grave lesions of the maxilla. The former, however, is frequently seen, and occurs as the result of the congenital absence of certain tooth follicles, or of the compression they are subjected to under certain circumstances. As an instance of this last variety may be mentioned the absence of the lower wisdom tooth, which is wanting in many persons. This form of anomaly does not, however, present any practical interest. The same cannot be said of the two other forms, *the early eruption and the tardy eruption.* The appearance of the teeth at, or very shortly after birth, may be accompanied by a series of accidents necessitating medical aid, as in a case previously reported by myself, in which the extraction of two teeth, cut on the second day after birth, was followed by intractable hemorrhage, and by the death of the child. On the other hand, the eruption of the teeth at an advanced age is often attended with grave complications: compression of the dental arch, abscesses, tumors, &c. The two following cases, which occurred recently in my private practice, appear to me to be of sufficient interest to be recorded.

1. *Premature eruption in a new-born child.*—Case: Two lower middle incisors cut at birth; extraction; incontrollable hemorrhage; death. Mrs. X., primipara, well developed woman, was delivered on the 7th of December, 1882, of a strong, healthy-looking male child, weighing seven pounds. On the very day of its birth the midwife noticed that the child bore in the centre of the lower gum two small teeth, which were very loose. Without asking the medical attendant's advice she pushed them out with her finger, very easily she says. Hemorrhage set in during the night At first it was quite superficial and very slight. It increased, however, and notwithstanding every means employed to check it: compression, applications of alum, plugging, perchloride of iron, and finally the actual cautery. The hemorrhage continued for six days, when the child died perfectly exsanguine.

The first point to be considered in these cases is the interpretation of the premature eruption of the teeth. Have we to deal with early supernumerary teeth or with preternaturally early milk teeth? There can, I think, be no doubt in the matter. These teeth form part of the normal dentition; for if they happen to fail from any cause whatever, their places remain empty during the whole of the first dentition. It is therefore a great mistake to look upon these teeth as superfulous and useless, and upon their removal as attended with no evil results.

I venture to assert again—

1. That no interference of any kind should be allowed in the case of the early eruption of milk teeth.

2. That in most cases these teeth may and do regain a certain degree of firmness, notwithstanding the short size of their roots.

3. That the only serious inconvenience caused by them is the difficulty in nursing and that this may be remedied by the use of artificial teats.

4. That a sufficient reason for abstaining from any surgical interference is the possibility of accidents, and especially of fatal hemorrhage.

II. *Late eruption in the aged.*—Case: Appearance in a woman, aged 76, of a tumor in the palate, of doubtful nature at first; intense and persistent neuralgia; permanent canine, which had never been cut, appearing in the centre of the tumor; sudden cessation of all symptoms.

Mrs. B., aged 66, informs us, that about ten years ago a small, hard indolent tumor, appeared in the centre of the roof of the palate. She was wearing at the time an artificial set of teeth, and the swelling being thought to depend upon this, an incision was made into it. The tumor went on increasing, however, and soon gave rise to violent and persist neuralgic symptoms in the infra and supra orbital and temporal regions. No surgical measures were taken, though the tumor was at this time considered to be a chronic abscess in the vault of the palate. No internal remedies proved of any avail.

Two years ago Mrs. B. noticed that in the centre of the tumor in the palate. which had now increased to the size of a large nut, a sharp, hard point could be felt by the tongue and finger; and also that the neuralgic symptoms had passed off ever since its appearance.

On looking into the mouth it was easy to make out the existence of a partially erupted crown of a canine tooth, projecting about one line and a half beyond the mucous membrane, and lying to the right of the median line, a little more than one inch benind the alveolar border.

Mrs. B. having lost all her teeth more than ten years previously, she was thought to be an example of tertiary dentition; but on carefully cross-examining the patient, it was found that at the time of the completion of her secondary dention (between eighteen and twenty) a distinct space had remained between two of the teeth on the right side only, the arch being quite complete on the opposite side. We had evidently here to deal then with the permanent canine tooth which had failed to make its appearance at the proper time, and was cut at this advanced period of life.

This case bears out the opinion which I expressed in a former paper, that all these instances of the eruption of teeth at an unusually late age may be explained by the tardy eruption of normal teeth, and that the existence of a third or fourth dentition is anything but proved.

I have but very little to say regarding the practical bearings of this case. No surgical interference appeared justifiable in the present instance, owing *first* to the possible dangers of such a curse; *secondly*, to the difficulties attending the removal of a partially erupted palatine tooth; and *lastly*, to the absence of any troubles of any kind.—*British Dental Journal.*

